# Antibacterial Activity of Positively and Negatively Charged Hematite (*α*-Fe_2_O_3_) Nanoparticles to *Escherichia coli*, *Staphylococcus aureus* and *Vibrio fischeri*

**DOI:** 10.3390/nano11030652

**Published:** 2021-03-08

**Authors:** Svetlana Vihodceva, Andris Šutka, Mariliis Sihtmäe, Merilin Rosenberg, Maarja Otsus, Imbi Kurvet, Krisjanis Smits, Liga Bikse, Anne Kahru, Kaja Kasemets

**Affiliations:** 1 Research Laboratory of Functional Materials Technologies, Faculty of Materials Science and Applied Chemistry, Riga Technical University, Paula Valdena 3/7, LV-1048 Riga, Latvia; Andris.Sutka@rtu.lv; 2 Laboratory of Environmental Toxicology, National Institute of Chemical Physics and Biophysics, Akadeemia tee 23, 12618 Tallinn, Estonia; Mariliis.Sihtmae@kbfi.ee (M.S.); Merilin.Rosenberg@kbfi.ee (M.R.); Maarja.Otsus@kbfi.ee (M.O.); Imbi.Kurvet@kbfi.ee (I.K.); Kaja.Kasemets@kbfi.ee (K.K.); 3 Institute of Chemistry and Biotechnology, Tallinn University of Technology, Akadeemia tee 15, 12618 Tallinn, Estonia; 4 Institute of Solid State Physics, University of Latvia, Kengaraga 8, LV-1063 Riga, Latvia; smits@cfi.lu.lv (K.S.); lbikshe@gmail.com (L.B.); 5 Estonian Academy of Sciences, Kohtu 6, 10130 Tallinn, Estonia

**Keywords:** hematite, *α*-Fe_2_O_3_ nanoparticles, hydrothermal synthesis, surface charge, antibacterial, nano-bio interactions, environmental safety, confocal, MicrobeJ

## Abstract

In the current study, the antibacterial activity of positively and negatively charged spherical hematite (*α*-Fe_2_O_3_) nanoparticles (NPs) with primary size of 45 and 70 nm was evaluated against clinically relevant bacteria *Escherichia coli* (gram-negative) and *Staphylococcus aureus* (gram-positive) as well as against naturally bioluminescent bacteria *Vibrio fischeri* (an ecotoxicological model organism). *α*-Fe_2_O_3_ NPs were synthesized using a simple green hydrothermal method and the surface charge was altered via citrate coating. To minimize the interference of testing environment with NP’s physic-chemical properties, *E. coli* and *S. aureus* were exposed to NPs in deionized water for 30 min and 24 h, covering concentrations from 1 to 1000 mg/L. The growth inhibition was evaluated following the postexposure colony-forming ability of bacteria on toxicant-free agar plates. The positively charged *α*-Fe_2_O_3_ at concentrations from 100 mg/L upwards showed inhibitory activity towards *E. coli* already after 30 min of contact. Extending the exposure to 24 h caused total inhibition of growth at 100 mg/L. Bactericidal activity of positively charged hematite NPs against *S. aureus* was not observed up to 1000 mg/L. Differently from positively charged hematite NPs, negatively charged citrate-coated *α*-Fe_2_O_3_ NPs did not exhibit any antibacterial activity against *E. coli* and *S. aureus* even at 1000 mg/L. Confocal laser scanning microscopy and flow cytometer analysis showed that bacteria were more tightly associated with positively charged *α*-Fe_2_O_3_ NPs than with negatively charged citrate-coated *α*-Fe_2_O_3_ NPs. Moreover, the observed associations were more evident in the case of *E. coli* than *S. aureus*, being coherent with the toxicity results. *Vibrio fischeri* bioluminescence inhibition assays (exposure medium 2% NaCl) and colony forming ability on agar plates showed no (eco)toxicity of *α*-Fe_2_O_3_ (EC_50_ and MBC > 1000 mg/L).

## 1. Introduction

Due to the spread of antibiotic resistance among microbial pathogens, there is an increasing interest in metallic nanoparticles (NPs) exhibiting antimicrobial activity [1,2]. The antimicrobial properties of NPs have been demonstrated towards both gram-positive and gram-negative bacteria, and they have been attributed to NP size, morphology, surface charge, catalytic, photoactive, optical, and thermal properties [3,4]. In addition, different types of bacteria demonstrate variable susceptibility to NPs [5,6]. The most efficient antimicrobial metal oxide NPs are CuO [7,8], Cu_2_O [7,9], Ag_2_O [10], Ag_4_O_4_ [11,12], ZnO [1,13], TiO_2_ [1,14], and also MgO [15,16]. In addition to potential antimicrobial applications, iron oxide NPs have gained attention for their use in biomedicine for targeted drug delivery, magnetic resonance imaging, and as possible anticancer agents [17,18,19,20,21,22,23]. 

Different iron oxide compounds can be found in nature: hematite (*α*-Fe_2_O_3_), maghemite (*γ*-Fe_2_O_3_) and magnetite (Fe_3_O_4_) [17,24,25]. Iron oxide NPs are easy to obtain; their synthesis is relatively cheap, and the methodology allows to develop them in a broad range of particle sizes [3,25,26,27,28,29]. Moreover, as mentioned above, iron oxides are abundant in nature [30]. Hematite (*α*-Fe_2_O_3_) is the most stable among iron oxides, as it is thermodynamically stable at ambient conditions, chemically stable at various pH [3,27,28,29], and can be synthesized by simple aqueous (green) approaches [31,32] in various shapes and with a broad size range [25]. Hematite NPs have gained a lot of attention due to their diverse applications, e.g., photoelectrochemical water splitting [33], as ingredients in sunscreens and cosmetics to optimize the present UV filter efficiency [34,35], and as anticancer [20,21,22,23] and water disinfection agents [36]. The production and application of NPs necessitate the safety evaluation of these materials that unfortunately always lags behind the respective technological developments [37]. As an example, the information on the ecotoxicity of iron oxide NPs started to emerge just about ten years ago in 2009 [38]. 

According to the (eco)toxicological database NanoE-Tox [38], Fe-oxide NPs (i.e., Fe_2_O_3_ and Fe_3_O_4_ taken together as FeOx) demonstrated the lowest toxicity within seven types of NPs evaluated: Ag > ZnO > CuO > CeO_2_ > CNTs > TiO_2_ > FeOx. In addition, the share of toxicity data for FeOx NPs (1518 toxicity values altogether) was about 10-fold lower than for TiO_2_ NPs and Ag NPs; it should be noted that up until 2015 there were no data about the possible toxicity mechanism of action of FeOx NPs. In addition, Rajendran et al. [39] showed that hematite NPs were not acutely toxic to mice up to an oral dose of 2 g/kg and also not acutely toxic to aquatic crustaceans *Artemia salina* up to 10,000 mg/L. The antifungal activity of hematite NPs towards *Saccharomyces cerevisiae* was studied using the inhibition of O_2_ consumption as a toxicity endpoint: No toxic effect was observed up to 1000 mg/L [40]. These data are coherent with the statement of the low toxicity of iron oxide NPs stated above.

During the past decade, iron oxide’s antimicrobial activity [3,5,41,42,43,44,45,46,47,48,49] and the respective mechanism of action have been studied. Analogously to ecotoxic properties of iron oxide NPs, their antimicrobial properties are not comparable to powerful antimicrobials such as copper and silver compounds that act at a few mg/L levels [38]. It has even been stated that iron oxide does not have antimicrobial properties [47,48]. However, there is a quite strong evidence in the literature on the antimicrobial activity of iron oxides, although the activity is rather weak (i.e., quite high concentrations of compounds are needed to gain antimicrobial effects). For example, Gabrielyan et al. [5] demonstrated moderate antimicrobial activity of magnetite Fe_3_O_4_ NPs upon exposure of bacteria to 500 mg/L of NPs that led to ~2.2-fold decrease in the growth rate of *E. coli* and ~1.5-fold decrease of the growth rate of *Enterococcus hirae*. Thus, there was more strong effect to *E. coli* (a gram-negative bacterium) than to *E. hirae* (a gram-positive bacterium), probably due to the differences in the bacterial cell wall structure. The same authors in their following paper reported more remarkable antibacterial effects of citric acid coated magnetite Fe_3_O_4_ NPs towards *E. coli*: 250 mg/L of citric acid coated Fe_3_O_4_ NPs inhibited the growth of *E. coli* by 2-fold (a bacteriostatic effect), whereas the kanamycin resistant *E. coli* was inhibited slightly more at up to 3-fold [43]. Almost similar results (MIC = 150 mg/L) were reported for hematite Fe_2_O_3_ NPs towards *E. coli* and *Vibrio cholerae* [21]. Thus, although at relatively high concentrations, the iron oxide has antibacterial properties.

One of the possible bactericidal activity of iron oxide NPs is due to the generation of reactive oxygen species (ROS), which are free radicals that can harm the cells in multiple ways, e.g., breaking of DNA strands as well as the inactivation of enzymes and peroxidation of membrane lipids causing the death of bacteria [3,41]. Arakha et al. [41] showed that positively charged chitosan-coated iron oxide (Fe_3_O_4_/γ-Fe_2_O_3_) NPs attracted negatively charged bacterial surface, leading to enhanced ROS production in the close vicinity of bacterial surface (see below). In addition, in the case of iron oxide NPs, ROS production follows the Fenton reaction: H_2_O_2_ causing DNA and protein damage is routinely produced in all aerobic organisms as a by-product of their respiration [3,41]. However, the intracellularly produced H_2_O_2_ is counterbalanced by various scavenging enzymes like superoxide dismutase present in the cells. That may lead to different redox reactions known as Fenton reaction involving Fe^3+^ and Fe^2+^, resulting in the generation of various radicals that are more potent ROS than H_2_O_2_ [41]. Specifically, when the H_2_O_2_ reacts with free ferrous ions via Fenton reaction, highly reactive •OH are formed [50]. Chan et al. [29] showed that Fe^2+^/Fe^3+^ conversion with H_2_O_2_ mainly occurs on the surface of iron oxide crystals rather than in the solution. Arakha et al. [41] showed that in the case of magnetic iron oxide (Fe_3_O_4_/γ-Fe_2_O_3_) NPs (50 μM), the ROS formed in the redox process on the surface of NPs supported by the interaction of positively charged (chitosan-coated) iron oxide NPs with bacterial surface depolarized the bacterial membranes, causing membrane damage and cell death. Analogous effects were observed by Zhang et al. [44,45], who showed that upon exposure of *E. coli* to hematite NPs (100 mg Fe/L), progressive sorption of NPs on *E. coli* cells’ surface led to decreased bacterial viability, probably due to deformation of cells and disruption of surface appendages (flagella) as proven by scanning electron microscopy (SEM) and atomic force microscopy (AFM) images.

There is no consensus on the toxicity and proposed mechanism of toxicity of iron oxides and hematite (Table 1) despite numerous literature reports. This can be partly due to variation in experimental details and testing media, but it can also be due to the variation in NPs characteristics and interaction with assays, which can affect the obtained results and contribute to the differences among the literature reports [1].

The present paper describes a simple green method for the synthesis of *α*-Fe_2_O_3_ NPs. To control the surface charge on *α*-Fe_2_O_3_ NPs to yield positive and negative values while preserving their size and colloidal behavior, the coating technique utilizing charged organic molecules have been applied. Citrate is a biocompatible molecule and has carboxylate groups that allows it to give to initially positively charged *α*-Fe_2_O_3_ surface a strong negative charge [51]. The antibacterial effect of *α*-Fe_2_O_3_ having positive and negative surface charges was evaluated against gram-positive bacterium *S. aureus* and gram-negative bacterium *E. coli*. As typical bacterial culture media can potentially modify iron oxides’ physicochemical properties, NP−cell interactions, and toxicity in antibacterial assays [8,46,49,52,53], tests were performed in deionized water (DI). 

Additionally, the ecotoxicity of the prepared NPs to bioluminescent marine gram-negative bacterium *V. fischeri* was evaluated. The *α*-Fe_2_O_3_ NPs potential to produce ROS was evaluated in abiotic and biotic conditions. The effect of the NPs surface charge on NP-cell interactions was evaluated by confocal laser scanning microscopy (CLSM) as well as by flow cytometry. To our knowledge, it is the first time that CLSM in reflective mode combined with MicrobeJ association analysis has been applied to study possible cellular interactions with positively charged and negatively charged NPs, and this approach can help to widen the methodological toolbox of nanotoxicology. 

## 2. Materials and Methods 

### 2.1. Materials

Iron (III) nitrate nonahydrate (Fe(NO_3_)_3_∙9H_2_O, ≥98%, Steinheim, Germany), sodium chloride (NaCl, ≥99%, Steinheim, Germany), trisodium citrate (C_6_H_5_Na_3_O_7,_ Steinheim, Germany), 2′, 7′ Dichlorofluorescin diacetate (C_24_H_16_Cl_2_O_7_, ≥98%, Jerusalemci, Israel) and hydrogen peroxide solution (H_2_O_2_, 30%, Steinheim, Germany) were purchased from Sigma Aldrich and used as received. High purity deionized (DI) water obtained from a Milli-Q^®^ system (Merck Millipore, Darmstadt, Germany) was used for all the tests. Mueller–Hinton agar (MHA) was purchased from Sigma Aldrich (Bangalore, India). Tryptone, yeast extract, and agar were obtained from LabM (Lancashire, UK). 

### 2.2. Synthesis of the Hematite (α-Fe_2_O_3_) NPs

*α*-Fe_2_O_3_ NPs with positive surface charge (*α*-Fe_2_O_3_-**p**) were synthesized by the aqueous hydrothermal method. Briefly, aqueous solutions of 0.05 M and 0.1 M iron (III) nitrate were vigorously stirred at room temperature for 30 min, poured into a 50 mL Teflon-sealed stainless-steel autoclave, and hydrothermally treated at 150 °C for 24 h. After completion of the reaction, the autoclave was cooled to room temperature naturally. The red precipitates were collected and washed several times with DI water until reaching neutral pH and then dried at 80 °C. The surface of *α*-Fe_2_O_3_ NPs was modified to negative surface charge (*α*-Fe_2_O_3_-**n**) using aqueous 0.1 M trisodium citrate (TSC) solution, stirred overnight at 80 °C, washed several times with DI water until neutral pH and dried at 80 °C.

### 2.3. Physicochemical Characterization of the Hematite (α-Fe_2_O_3_) NPs

Crystalline phases of the synthesized NPs were analyzed by Rigaku MiniFlex X-ray diffractometer (XRD, Tokyo, Japan) with Cu Kα (λ 1.540593 Å) radiation in the scan range 5–90°. The morphology and size of synthesized NPs were characterized using a scanning electron microscope (SEM, Lyra, Tescan and Helios 5UX, FEI, Eindhoven, Netherland) and transmission electron microscopy (TEM, Tecnai F20, FEI, Eindhoven, Netherland). Average hydrodynamic diameter (dh) and zeta (ζ) potential of NPs (50 mg/L) in DI water and 2% NaCl solution were measured using Zetasizer Nano ZS (Malvern Instruments Ltd, Worcestershire, UK). The dh and ζ-potential were measured in 1mL DTS1061 folded capillary cell (Malvern Instruments, Worcestershire, UK) immediately after preparation (0 h) and after 24 h. Attenuated total reflection–Fourier transform infrared (ATR-FTIR, Varian 800 FT-IR, Scimitar Series, Fitchburg, WI, USA) measurements were recorded on a Attenuated Total Reflectance (ATR, GladiATR^TM^, Pike technologies, Fitchburg, WI, USA) mode in the range of 4000 to 400 cm^−1^ at a resolution of 4 cm^−1^ after 50 continuous scans.

#### Analysis of the Dissolved Fraction of Hematite NPs

To mimic the dissolution of NPs in DI (a testing medium for *E. coli* and *S. aureus* assays), NPs suspensions in DI water (100 mg/L) were prepared and incubated for 30 min and 24 h as described in Section 2.4. Then, 3 mL of each suspension was centrifuged for 20 min at 9500 rpm, and 50 µL of the supernatant was carefully removed and mixed with gallium (Ga) internal standard in the ratio of 1:1. The concentration of the metals in the supernatant was analyzed by total reflection X-ray fluorescence spectroscopy (TRXF) using Picofox S2 (Bruker AXS Microanalysis GmbH, Karlsruhe, Germany, a detection limit of Fe 1 ppb). The concentration of metals was quantified with the Spectra software (AXS Microanalysis GmbH, Karlsruhe, Germany).

### 2.4. Spot Test for Estimating Minimal Bactericidal Concentrations (MBCs)

The antibacterial efficiency of NPs was evaluated using a “spot” assay as described in Kasemets et al. [63] and Suppi et al. [64] by estimating the MBC of the tested NPs against gram-negative bacteria *E. coli* MG 1655 (obtained from the *E. coli* genetic stock center, Yale University) and gram-positive bacteria *S. aureus* ATCC 6538 (obtained from the American Type Culture Collection, ATCC). Briefly, test bacteria were exposed to NPs in DI water in 96-well microplates at room temperature. After 30 min and 24 h exposure, 3 µL of the cell suspension was pipetted as a “spot” onto the Luria–Bertani (LB) agar medium, and the seeded agar plates were incubated for 24 h at 30 °C. This measures the ability of the test bacteria to grow on a toxicant-free agar growth medium, i.e., the formation of a visible “spot” (colonies) was used as a toxicity endpoint. MBC was determined as the lowest tested nominal concentration of NPs which completely inhibited the formation of visible colonies of bacteria on agar medium.

The stock suspensions of *α*-Fe_2_O_3_ (2 g/L) were prepared in DI water and homogenized using an ultrasonic probe (Branson Digital Sonifier^®^, Danbury, CT, USA) at 40 W for 3 min once after preparation. Before the spot test, the stocks were diluted in DI water to obtain the test concentrations. All NPs were tested in following nominal concentrations: 1, 10, 100, 250, 500, and 1000 mg/L.

### 2.5. Agar Diffusion Test

The agar diffusion test was performed to evaluate the possible release of toxic compounds from the *α*-Fe_2_O_3_ NPs. Here, 100 µL of *E. coli* and *S. aureus* bacteria (~10^6^ CFU/mL) were swabbed onto MHA plates using a sterilized cotton bud. Next, 100 µL of NP suspensions of 10–1000 mg/L were pipetted into the wells made in agar, and MHA plates were incubated at 30 °C for 24 h. The compound’s release was investigated by the formation of growth inhibition zones of bacteria (a clear zone).

### 2.6. Flash Assay with Vibrio fischeri

The stock suspensions of tested NPs were prepared in 2% NaCl; the 2% NaCl served as a control and diluent solution for all the samples. A kinetic *V. fischeri* bioluminescence inhibition test (“A Flash assay”) was performed at room temperature (ca. 20 °C) in 96-well white polypropylene microplates, following the ISO 21338:2010 protocol (water quality kinetic determination of the inhibitory effects of sediment, other solids, and color samples on the light emission of *V. fischeri*). A freeze-dried *V. fischeri* reagent (Aboatox, Turku, Finland) was used. Briefly, 100 µL of bacterial suspension was added to 100 µL of the tested compound in the microplate well by automatic dispensing. Bacterial luminescence was continuously recorded during the first 15 s after dispensing (no additional mixing of the sample) and once again after 30 min incubation. The Microplate Luminometer Orion II (Berthold Detection Systems, Pforzheim, Germany), controlled by the Simplicity version 4.2 Software, was used for the dispensing and recording of the luminescence. In each measurement series, both negative (2% NaCl) and positive (3,5-dichlorophenol) controls were included.

After 1 and 24 h of exposure to the NPs or controls (exposure time was counted from the beginning of Flash assay), 3 µL of the cell suspension from each microplate well was pipetted as a spot onto an Beneckea Harvey (BH) agar medium containing (per L): yeast extract 3 g, tryptone 5 g, glycerol (99%) 2 mL, NaCl 30 g Na_2_HPO_4_*12H_2_O 9.45 g, KH_2_PO_4_ 1 g, (NH_4_)_2_HPO_4_ 0.5 g, MgSO_4_*7H_2_O 0.3 g, agar 15 g. The inoculated agar plates were incubated for 48 h at room temperature in the dark, and the MBC of the tested NPs was determined visually. 

### 2.7. Abiotic and Biotic ROS Measurement

An analysis of NPs-induced ROS in abiotic conditions was performed using a fluorescent dye 2′,7′-dichlorofluorescein diacetate (DCF-DA) essentially as described in Aruoja et al. [65]. Briefly, 450 µL of DCF-DA (dissolved in ethanol at 1.3 mM) was freshly deacetylated to DCF by reacting with 1800 mL of 0.01 M NaOH for 30 min in the dark. The reaction was stopped by adding 9 mL of 25 mM sodium phosphate buffer (pH 7.4) to prepare a 52 μM DCF solution. The mixture was immediately placed on ice and protected from the light until use. After that, 100 μL of a NPs suspension and 100 µL of DCF solution were pipetted to each well of a 96-well black microplate. As a positive control, the Mn_3_O_4_ NPs were used. 

The potential of intracellular ROS formation (i.e., in the biotic conditions) by *α*-Fe_2_O_3_ NPs was assessed using DCF-DA as described in Käosaar et al. [66]. DCF-DA is a marker for total ROS production and a qualitative marker of oxidative stress in bacterial cells [67].

Briefly, bacterial suspensions from exponentially growing culture were exposed to NPs and positive control (H_2_O_2_) in DI water at room temperature for 30 min and 24 h in the dark on black 96-well polypropylene microplates. In this test, NPs and H_2_O_2_ were studied at the concentrations of 1–1000 mg/L. The ~10^6^ CFU/mL bacteria cell culture in DI water was prepared as for the cell viability test (see Section 2.4.), and 100 µL of cell suspension was added to the wells of the microplates containing 100 µL of the NPs suspensions in DI water. After incubation, 50 µL of the DCF-DA working solution (250 µg/mL in 25 mM Na-phosphate buffer, pH 7.2) was added to the samples in the wells of the microplate and incubated for 30 min and 24 h at room temperature. 

Fluorescence was recorded at excitation and emission wavelengths of 485 nm and 527 nm, respectively, using the Fluoroskan Ascent FL microplate reader (Thermo Labsystems, Finland). ROS levels in bacterial cells were calculated as fold-increase in fluorescence signal compared to the nonexposed bacteria. Results are given as the mean of three experiments ± SD. 

The abiotic and biotic ROS level was calculated as follows:

(1) $$F=\frac{F_{t30\;\left(sample\right)}}{F_{t30\;\left(control\right)}}$$

where *F_t_*_30_ (*sample*) is the fluorescence of the NP suspension in DI water (*t* = 30 min) after incubation with the fluorescent dye; and *F_t_*_30_ (*control*) is the fluorescence of blank DI water (*t* = 30 min) after incubation with the fluorescent dye. Fluorescence is presented in relative fluorescence units (RFU).

### 2.8. Flow Cytometry

A flow cytometry analysis was performed to analyze the interaction of *α*-Fe_2_O_3_ NPs with bacterial cells essentially as described by Feng et al. [68]. Briefly, exponentially growing *E. coli* and *S. aureus* cells were prepared as described in the Supplementary Material; they were diluted to the density OD_600nm_ = 0.4 (equals to 2 × 10^7^ CFU/mL for *E. coli* MG 1655 and 10^8^ CFU/mL for *S. aureus* ATCC 6538) and exposed to positively and negatively charged hematite NPs (designated as *α*-Fe_2_O_3_-**p**45 and *α*-Fe_2_O_3_-**n**45, respectively) at 50 mg/L for 30 min without shaking. Here, 70% ethanol was used as a positive control to obtain nonviable cells to determine the possible increase in the cell granularity due to the cell wall/membrane damage. Incubation of bacteria in ethanol was performed in the same conditions as for the test samples. Following the incubation, the bacteria were stained for 15 min with 5 μM Syto9 (Invitrogen S34854) in the dark at room temperature and analyzed by a BD Accuri™ C6 flow cytometer (BD Biosciences, Ann Arbor, MI, USA) using a 488 nm excitation laser and 533 nm wavelength filter. A total of 20 000 cells were analyzed for each sample. The percentage of bacterial cells interacting with hematite NPs was quantified by the shift in the side-scattered light (SSC being proportional to cell granularity) compared to control cells.

### 2.9. Confocal Laser Scanning Microscopy (CLSM) Analysis and Signal Quantitation

For the visualization of bacterial interactions with the tested NPs, 10 µL of NPs-exposed bacterial suspensions or nontreated controls were mixed with 10 µM Syto9 nucleic acid stain (final concentration 5 µM), incubated at room temperature for 10 min, spread on the microscopy slide, and dried at 37 °C for 15 min. Dry samples were submerged in a Mowiol mounting medium, covered by cover-glass and visualized with Zeiss LSM800 CLSM (Zeiss, Jena, Germany) using excitation/emission track settings of 488 nm/505–550 nm (to visualize Syto9-stained cells) and reflection mode at 640 nm (to visualize the NPs). The acquired three-dimensional multichannel images (at least 10 per slide) were converted into multichannel maximal projections using the Zeiss software interface and further analyzed by MicrobeJ software [69]—a plugin application for the open-source platform ImageJ [70]. Bacterial cells and NPs reflection signals (point foci) were detected and counted based on fluorescence intensity threshold in their respective channels. Reflection signals were counted as cell-associated if their center point localized closer than 400 nm to the closest cell boundary on CLSM maximal projection. The latter distance was empirically set, considering that the bacterial cells were slightly larger than the detected cell boundary based on Syto9 nucleic acid signal and that the reflection intensity maximum (point foci) of larger reflection signals appeared further from the cell boundary. All reflection signals were associated with no more than one parent cell. Statistically significant differences between the counts were analyzed using one-way ANOVA followed by Tukey’s post-hoc test at α = 0.05 for multiple comparisons in GraphPad Prism 8.3.0.

## 3. Results and Discussions

### 3.1. Synthesis and Physicochemical Characterization of the Hematite (α-Fe_2_O_3_) NPs

Two precursor concentrations (0.05 M and 0.1 M Fe(III) nitrate) were used for hematite (*α*-Fe_2_O_3_) NPs synthesis, yielding roughly two sizes of positively charged NPs, i.e., 45 nm and 70 nm, respectively. Both of these variants were coated with citrate to provide a negative surface charge. These 4 hematite NPs were used for further studies and designated as shown in Table 2.

#### 3.1.1. XRD and FTIR Analysis of the Synthesized NPs

**Figure 1:**
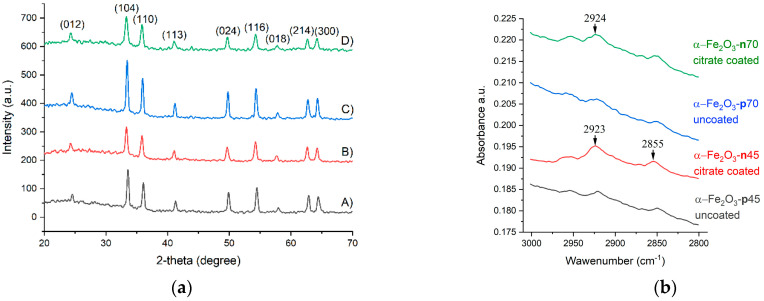
(**a**) XRD patterns of *α*-Fe_2_O_3_ nanoparticles (NPs): (A) *α*-Fe_2_O_3_-**p**45, (B) *α*-Fe_2_O_3_-**p**70, (C) *α*-Fe_2_O_3_-**n**45, (D) *α*-Fe_2_O_3_-**n**70. The peak positions of the pure rhombohedral phase of *α*-Fe_2_O_3_ (ICDD 98-000-0240). (**b**) Attenuated total reflection–Fourier transform infrared (ATR-FTIR) spectra of *α*-Fe_2_O_3_ NPs before and after citrate coating.

The XRD studies indicate that the hematite phase was successfully obtained by aqueous hydrothermal synthesis. All the diffraction peaks in the XRD pattern (Figure 1a) of all synthesized samples correspond to the hematite rhombohedral phase of *α*-Fe_2_O_3_ (ICDD 98-000-0240) with a space group R3c (No. 167). The hematite phase formed directly in the hydrothermal synthesis step without additional heating needed. The citrate modification step did not cause a change in the phase composition. 

The ATR-FTIR results confirm the formation of a citrate layer on *α*-Fe_2_O_3_ NPs after surface modification. This is indicated by the appearance of new vibrational bands at 2923, 2924, and 2855 cm^−1^ [71,72].

#### 3.1.2. SEM Analysis of the Synthesized NPs

**Figure 2:**
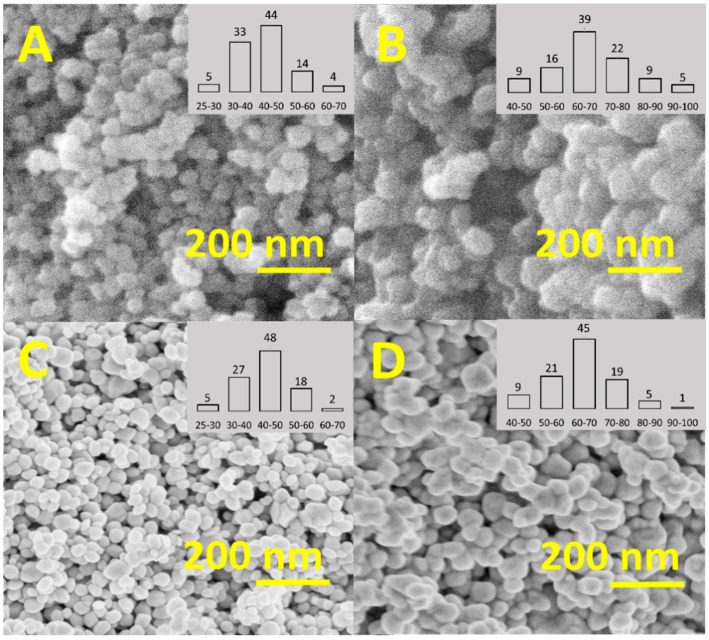
SEM observation of *α*-Fe_2_O_3_ NPs prepared with different concentrations of Fe (III) nitrate, (**A**) 0.05 M (*α*-Fe_2_O_3_-**p**45), (**B**) 0.1 M (*α*-Fe_2_O_3_-**p**70), and after ɑ-Fe_2_O_3_ NPs coating with the citrate, (**C**) 0.05 M (*α*-Fe_2_O_3_-**n**45), (**D**) 0.1 M (*α*-Fe_2_O_3_-**n**70).

The NPs morphology and size were studied using SEM, and the size distribution of the NPs was estimated by measuring the diameters of 100 NPs. Figure 2 presents the SEM images of the *α*-Fe_2_O_3_-**p** (A,B) and *α*-Fe_2_O_3_-**n** (C,D), and it shows that all the NPs had a spherical shape. According to the SEM, the size of the NPs increased from ~45 nm to ~70 nm by increasing the amount of the Fe (III) nitrate precursor from 0.05 M (A,C) to 0.1 M (B,D). The increase in size can be explained by the increased concentration of the monomer (growth species in affixed reaction volume) [73]. After the NPs were coated with citrate (Figure 2), the average size for the *α*-Fe_2_O_3_-**n** 0.05 M (C) and 0.1 M (D) remained unchanged.

#### 3.1.3. TEM Analysis of the Synthesized NPs

**Figure 3:**
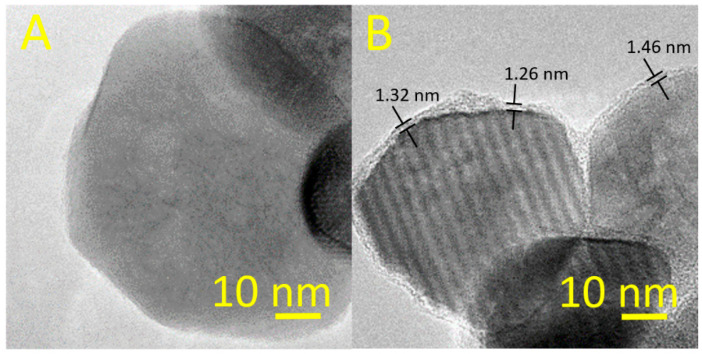
TEM images of the *α*-Fe_2_O_3_ NPs: (**A**) uncoated-positively charged (*α*-Fe_2_O_3_-**p**70), (**B**) citrate coated-negatively charged (*α*-Fe_2_O_3_-**n**70).

Figure 3 shows the TEM images of the surface of *α*-Fe_2_O_3_ NPs in bare form (left) and after citrate coating (right). TEM results confirm the formation of the citrate coating on the surface of *α*-Fe_2_O_3_ NPs. More additional information is found in the Supplementary Material.

#### 3.1.4. Analysis of the Hydrodynamic Size and ζ –Potential of Synthesized NPs

**Figure 4:**
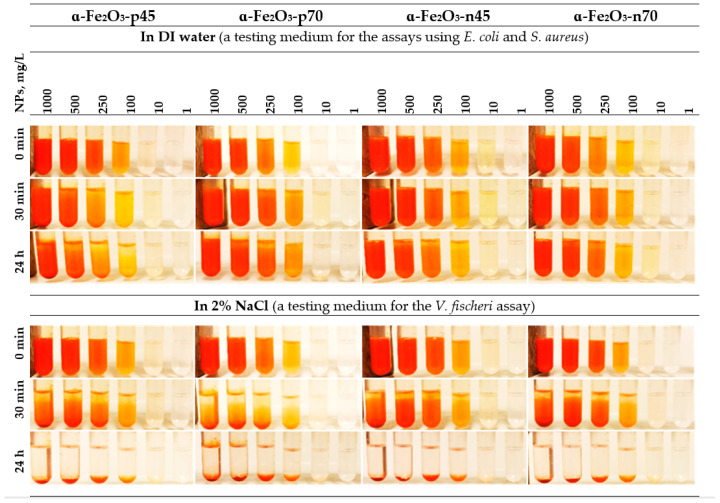
Visualization of stability of the *α*-Fe_2_O_3_ NPs in DI water and 2% NaCl (see also Table 3).

The ζ-potential (0 h) of *α*-Fe_2_O_3_-**p**45 and *α*-Fe_2_O_3_-**p**70 in DI water was +38.82 ± 0.9 mV and +40.25 ± 1.1 mV, respectively (Table 3). After citrate coating, these values shifted to −33.75 and −44.72 for *α*-Fe_2_O_3_-**n**45 and *α*-Fe_2_O_3_-**n**70, respectively (Table 3). The polydispersity indices (Pdl) after 24 h incubation in DI water were not exceeding 0.25 for the studied NPs (Table 3 and Figure 4), suggesting that the NPs suspensions had a narrow distribution and are stable [74,75]. After NPs were incubated in the 2% NaCl testing medium: aggregation, sedimentation and positive ζ-potential conversion to negative of the studied NPs were observed (Table 3 and Figure 4). The pH values of the NPs suspensions in DI water and the 2% NaCl testing media can be found in the Supplementary Material (Table S1).

#### 3.1.5. NPs Solubility in DI Water 

Hematite itself is relatively chemically inert and does not practically release Fe-ions [45]; this was also confirmed by the solubility test made in the current study, where at 100 mg/L the share of solubilized iron did not exceed 0.1 ppm (Table 3). Thus, shedding of the Fe ions in the amount that could cause the toxicity to bacteria was not observed. In addition, citrate coating had no effect on the NPs’ solubility (Table 3).

### 3.2. Antibacterial Activity

The results of the spot test showed that positively charged uncoated NPs were inhibiting the growth of *E. coli* at a concentration of 100 mg/L already after a 30 min exposure, whereas the toxicity of the NPs was increasing in time where inhibitory effects were evident already at 10 mg/L and the 24 h MBC value was 100 mg/L. The effect was not dependent on the particle’s size. The negatively charged citrate-coated NPs had no inhibitory effect up to the highest tested concentration, i.e., 1000 mg/L (Figure 4, upper panels).

**Figure 5:**
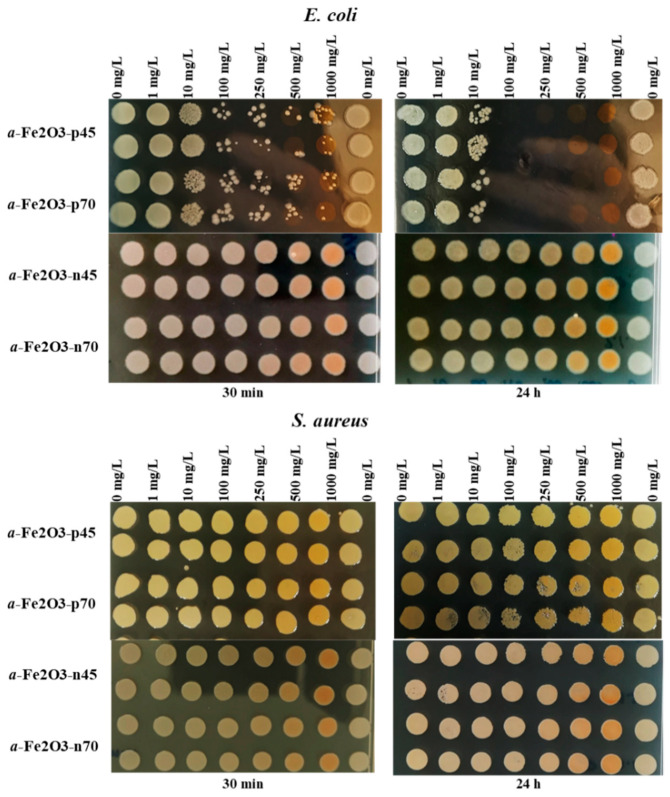
Viability of *Escherichia coli* (upper panels) and *Staphylococcus aureus* (lower panels) after exposure to *α*-Fe_2_O_3_ NPs in DI water for 30 min and 24 h at room temperature. Viability was evaluated by the ability of exposed bacteria to yield colonies at the nutrient agar plate as indicated on the panels.

The hematite NPs, irrespective of the size and surface charge, had no inhibitory effect towards *S. aureus* up to the highest tested concentration, i.e., MBC > 1000 mg/L (Figure 5, lower panels).

Abbaszadegan et al. [4] demonstrated that *S. aureus* and *E. coli* were more sensitive to Ag NPs with a positive charge, but *S. aureus* was more sensitive to Ag NPs with negative charge compared with *E. coli*. Schwegmann et al. [46] showed that the NP surface charge (zeta potential varied by the changes of the testing medium pH) influenced the sorption and toxicity of magnetic iron oxide (Fe_3_O_4_/γ-Fe_2_O_3_) NPs on bacteria *E. coli* and yeast *S. cerevisiae*. At pH 4 (which provides positive zeta potential), a strong bactericidal effect of iron oxide NPs on E. coli was observed, in comparison with pH 10 (which provides negative zeta potential) where a small killing effect occurred.

### 3.3. Agar Diffusion Test

**Figure 6:**
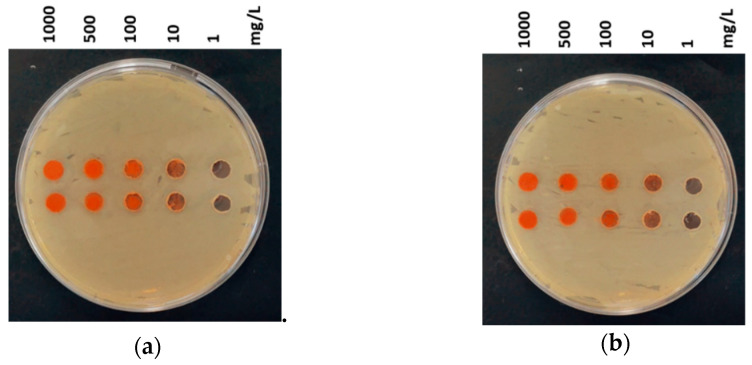
Agar diffusion test using *Escherichia coli*: (**a**) *α*-Fe_2_O_3_-**p**45 (**b**) *α*-Fe_2_O_3_-**n**45. Exposure time 24 h, temperature 30 °C, Mueller–Hinton agar medium (MHA). Agar diffusion test images for *Staphylococcus aureus* can be found in the Supplementary Material (Figure S2).

Formation of the growth inhibition zones as a result of compounds release was not observed (Figure 6), which correlates with the NPs’ solubility data (Table 3). 

### 3.4. Ecotoxicity 

The naturally luminescent bacterium *V. fischeri* is a useful tool to estimate the (eco)toxicity of different chemicals by measuring the inhibition of bacterial light production due to the adverse effects of toxicants on bacterial membranes that is dose-dependent and very rapid—the effects are detectable already after the first seconds of contact between the bacteria with the toxicant. In the flash assay, each sample is used as its own reference, and so color and turbidity correction is possible with minimal manipulation; this has been proved to be a suitable tool for the assessment of toxicity in solid and colorful samples [76,77,78]. The time scale and pattern of the kinetics of bacterial luminescence in response to certain NPs allows for a comparison of the toxic action of different NPs with respect to the disturbance of the cellular membrane integrity [76].

As *E. coli* and *V. fischeri* are both gram-negative bacteria with the similar cell wall structure, their response to most chemicals should theoretically be comparable [78]. Thus, the same interaction was expected between bacteria and positively charged NPs. Kurvet et al. [78] demonstrated that *V. fischeri* was more sensitive to Cu^2+^ and anilines compared with *E. coli*. According to the results of the current study, all four types of *α*-Fe_2_O_3_ NPs were not toxic to *V.* *fischeri* in the bioluminescence inhibition assay even at the highest tested concentration, i.e., 30 min EC_50_ > 1000 mg/L, showing that *α*-Fe_2_O_3_ was not (eco)toxic according to this test. Figure 6 shows the kinetics of the toxic effect of hematite NPs towards *V.* *fischeri* during 15 s of contact with NPs: Luminescence inhibition was not observed, but there was a clear shading effect of luminescence depending on hematite concentration. However, one of the reasons why toxicity was not observed could be the impact of 2% NaCl in the testing medium, as NPs aggregation, sedimentation, and positive ζ-potential conversion to negative were observed (Table 3 and Figure 4). Zhou et al. [79] supported this hypothesis by demonstrating in TEM and field emission scanning electron microscopy that the binding of Ag or AuNPs to *E. coli* bacteria was different depending on their aggregation state.

According to Mamindy-Pajanya Y. [80], the presence of hematite and zero-valent iron in sediments significantly reduced the toxicity of marine dredged sediments for *V. fischeri*. For iron oxide (Fe_3_O_4_) NPs, previously reported toxicity values for *V. fischeri* were >100 mg/L [65] and 240 mg/L [81].

**Figure 7:**
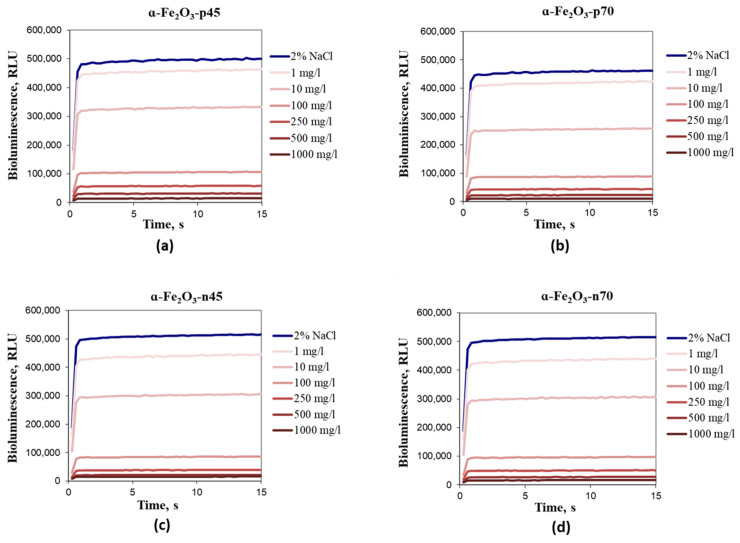
Kinetics of the *Vibrio fischeri* bioluminescence inhibition upon exposure of bacteria to different concentrations of *α*-Fe_2_O_3_ NPs. (**a**) *α*-Fe_2_O_3_-**p**45; (**b**) *α*-Fe_2_O_3_-**p**70; (**c**) *α*-Fe_2_O_3_-**n**45; (**d**) *α*-Fe_2_O_3_-**n**70

**Figure 8:**
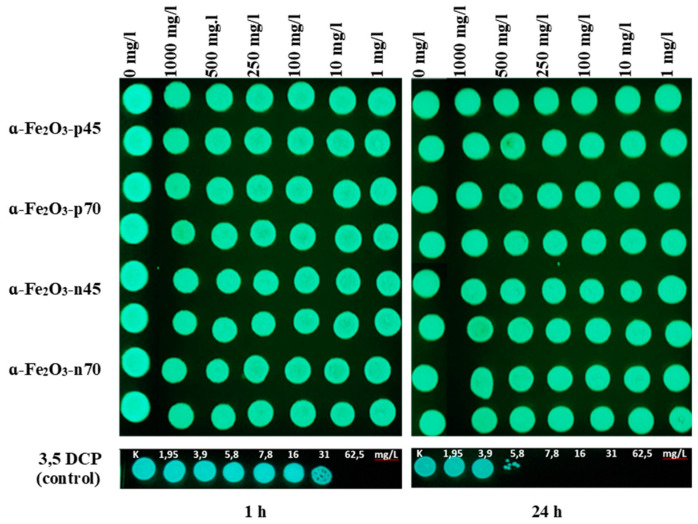
Viability of *Vibrio fischeri* after exposure to *α*-Fe_2_O_3_ NPs in 2% NaCl for 30 min and 24 h at room temperature. Viability was evaluated by the ability of exposed bacteria to yield colonies at the Beneckea Harvey (BH) agar plate as indicated on the panels. Blue-green spots are bioluminescent bacterial colonies photographed in the dark; 3,5-dichlorophenol (3,5 DCP) was used as a positive control.

As at hematite NP concentrations starting from 100 mg/L, luminescence shading was observed (Figure 7) as the NPs are very colorful (Figure 4), and therefore also a spot test was performed: After 1 and 24 h of incubation, no inhibitory effect of hematite NPs on colony forming ability of *V. fischeri* was shown (Figure 8).

### 3.5. Abiotic and Biotic Reactive Oxygen Species (ROS) 

**Figure 9:**
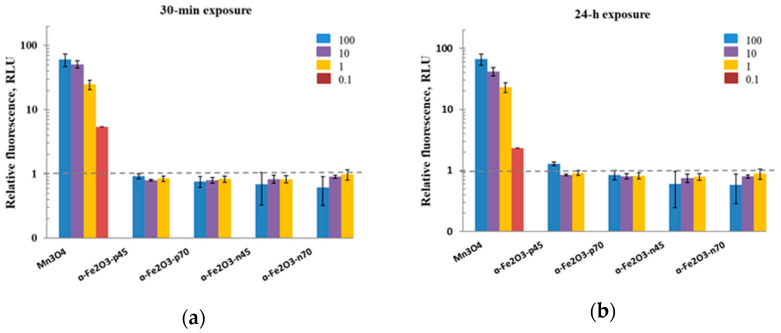
Generation of reactive oxygen species (ROS) measured with fluorescent dye DCF-DA in abiotic conditions (i.e., without of the cells) after 30 min (**a**) and 24 h (**b**) exposure to hematite NPs (1–100 mg/L), and Mn_3_O_4_ NPs—a positive control (0.1–100 mg/L)—in DI water. The values are presented as fold-increase in fluorescence in the presence of the NPs compared to the control (DI water); the mean of 4 experiments is given ± SD. All exposure concentrations are nominal. The dashed horizontal line indicates the ROS level in the DI water.

One of the possible antibacterial mechanisms of *α*-Fe_2_O_3_ NPs is the production of ROS [41,49,56]. Initially, the potential of *α*-Fe_2_O_3_ NPs to induce the ROS was studied in abiotic conditions, i.e., in DI water with no test organisms present. Suspensions of NPs in DI water were prepared in the same manner as for the cell viability test (see Section 2.4.). Based on the results, even the highest concentration of hematite NPs tested (100 mg/L) did not induce ROS in abiotic conditions compared to the negative control (DI water; see the dashed line in Figure 9); similar results were obtained for Fe_3_O_4_ NPs in the study by Aruoja et al. [65]. The assay worked correctly as demonstrated by the ROS produced by a positive control, i.e., the Mn_3_O_4_ NPs (see Figure 9 and also the study by Aruoja et al. [65]). It was not possible to test higher NPs concentrations (>250 mg/L) in this assay due to the colorful nature of NPs (Figure 4) and fluorescence shading.

Arakha et al. [41], in using DCFH-DA assay, demonstrated that iron oxide NPs (Fe_3_O_4_/γ-Fe_2_O_3_) caused intracellular ROS production in the *E. coli* and *B. subtilis* cells. Both negatively and positively charged iron oxide NPs resulted in a significant increase in the fluorescence intensity, with a relatively higher change in the presence of positively charged, chitosan-coated iron oxide NPs. The observation was used by the authors to explain why the positive iron oxide NPs had higher antibacterial activity than negative iron oxide NPs. Qu C. et al. [49] observed that *P. putida* exposure to 500 mg/L hematite (*α*-Fe_2_O_3_) NPs resulted in a 1.4- to 2.7-fold increase in the intracellular ROS concentrations. ROS content increased significantly with the increase in NP concentration but decreased with NP size increase. Ali K et al. [56] observed the increase of intracellular ROS production in pristine and low EPS (extracellular polymeric substances) *P. aeruginosa* cells as a result of the cellular uptake of ALE-*α*-Fe_2_O_3_ (synthesized with *Aloe vera* extract) and bare-*α*-Fe_2_O_3_ NPs. Furthermore, hydrogen peroxide (H_2_O_2_), is produced in all aerobic organisms during respiration [3,41]. This can lead to different redox reactions known as the Fenton reaction as already elaborated in the Introduction [29,41].

**Figure 10:**
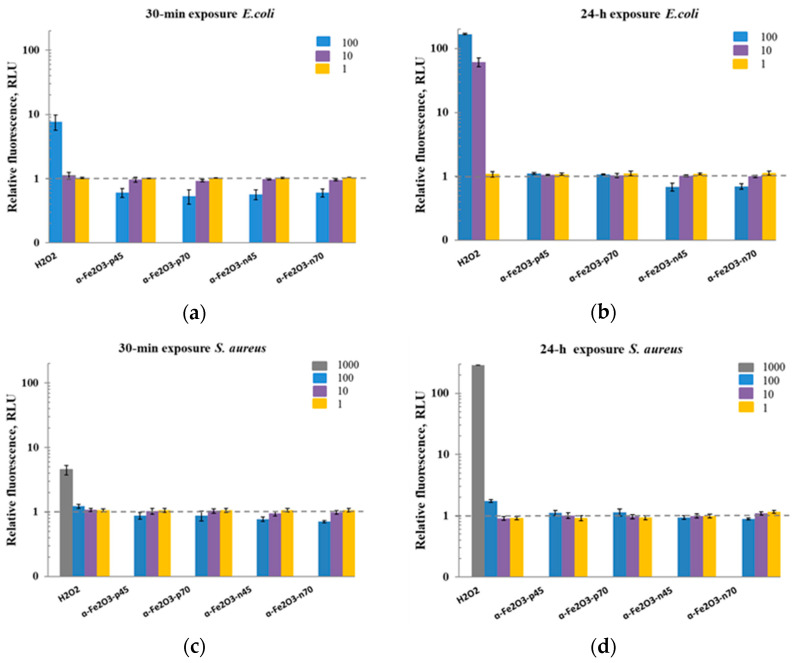
Generation of ROS measured with fluorescent dye DCF-DA in the biotic conditions after 30 min (**a**–**c**) and 24 h (**b**–**d**) exposure to hematite NPs (1–100 mg/L), and H_2_O_2_ as a positive control (1–100 mg/L for *Escherichia coli*; 1–1000 mg/L for *Staphylococcus aureus*) in DI water. The values are presented as fold-increase in fluorescence of exposed cells compared to unexposed cells; the mean of 3 experiments is given ± SD. All exposure concentrations are nominal. The dashed horizontal line highlights the ROS level in the control bacteria in DI water. Graphs of ROS generation in biotic conditions after 24 h of NP incubation with bacteria and 30 min after dye adding points can be found in the Supplementary Material (Figure S3).

Therefore, ROS generation in the presence of NPs was also studied in the biotic conditions, i.e., in DI water with the test organisms present. Although iron is known to generate intracellular ROS, driven by the Fenton reaction [29,82], neither positively nor negatively coated *α*-Fe_2_O_3_ NPs increased the fluorescence of DCF compared to the control, as indicated in Figure 10. Thus, ROS production was probably not involved in the antibacterial action of the studied *α*-Fe_2_O_3_ NPs. ROS production by added H_2_O_2_ in the presence of the *S. aureus* was also observed only at a higher concentration of 1000 mg/L; this is in the agreement with the toxicity results of H_2_O_2_ to *S. aureus* in Suppi et al. [64]. 

### 3.6. Assessment of the NPs Adhering to the Surface of Bacteria Cells by Confocal Laser Scanning Microscopy (CLSM) 

Possible interactions between the NPs and bacterial cells were visualized using CLSM in the reflection mode, which allows for the simultaneous detection of fluorescently stained bacteria and reflective signals of the metallic NPs [83].

**Figure 11:**
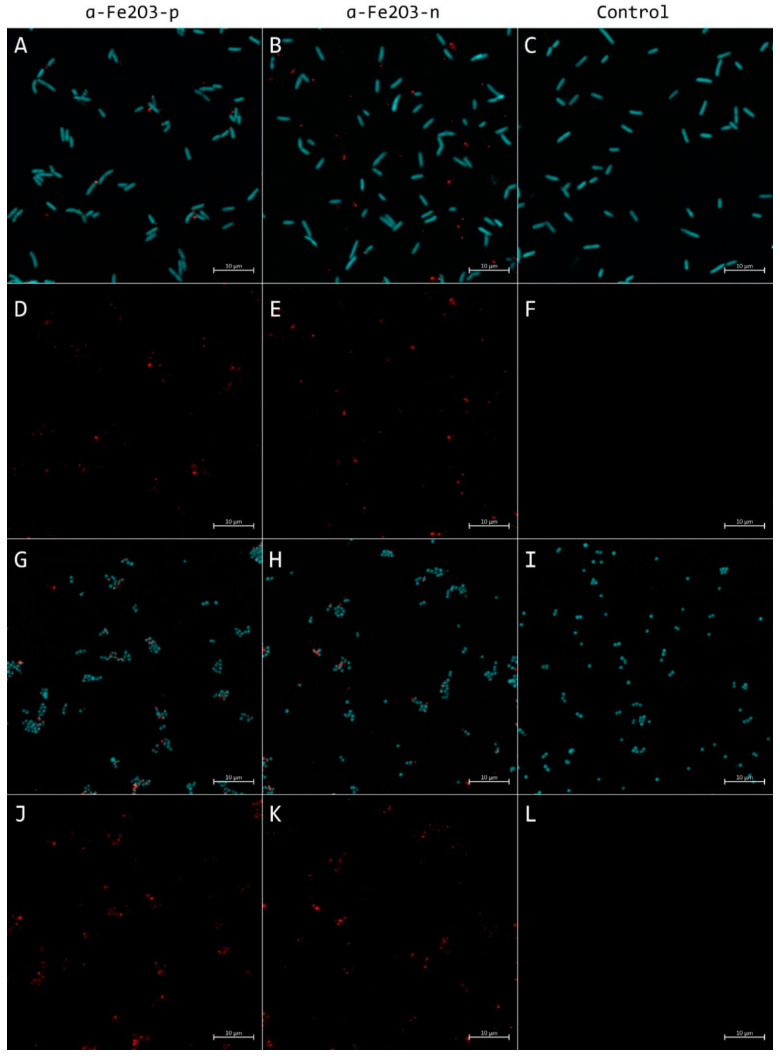
Representative maximal projections of confocal laser scanning microscopy (CLSM) images (pseudocolored: cellular Syto9 signal—cyan, NPs signal from reflection mode—red) of *Escherichia coli* (**A**–**F**) and *Staphylococcus aureus* (**G**–**L**) exposed to 100 mg/L *α*-Fe_2_O_3_-**p** (left column), *α*-Fe_2_O_3_-**n** (middle column) or incubated without NPs (right column) for 30 min. Merged multichannel images (**A**–**C**,**G**–**I**), as well as reflection channel signals, are presented (**D**–**F**,**J**–**L**). Scale bars represent 10 µm. Single-channel and multichannel projection images for 30 min and 24h time points can be found in the Supplementary Material (Figures S4 and S5). A respective quantitative analysis is presented in Figure 12.

**Figure 12:**
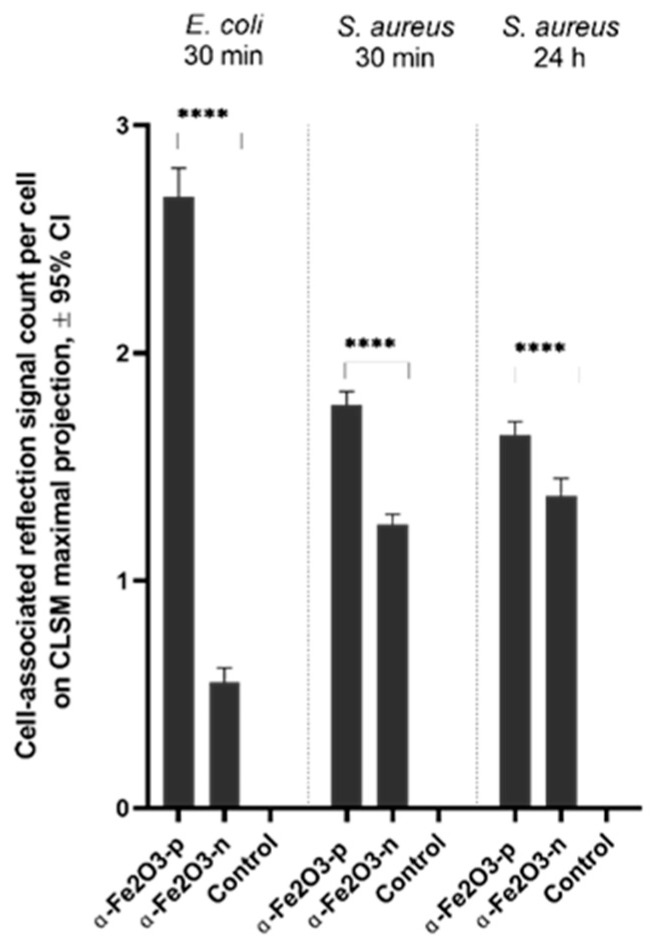
Results for quantitative cellular fluorescent and reflective NP signal analysis from maximal projections of CLSM images of bacteria exposed to 100 mg/L NPs or unexposed controls. Significantly more (*p* < 0.0001; ****) *α*-Fe_2_O_3_-**p** NPs signals were associated with both *Escherichia coli* and *Staphylococcus aureus* cellular signals than *α*-Fe_2_O_3_-**n** NPs signals, showing that more positively charged NPs were associated with generally negatively charged bacterial cells than negatively charged NPs and that this effect was larger for of *E. coli*, which is also in concordance with *α*-Fe_2_O_3_-**p** NPs being more toxic to *E. coli* than *S. aureus*. The 24 h time point was excluded from analysis for *E. coli* due to lethal exposure and thus poor cellular signals. No reflective NPs signals were detected in nonexposed controls.

CLSM images of *E. coli* and *S. aureus* exposed to *α*-Fe_2_O_3_ NPs showed the presence of NPs in the sample (Figure 11). After a 30 min exposure to 100 mg/L of *α*-Fe_2_O_3_-**p** NPs, the NPs could be seen at the cell perimeter of individual *E. coli* cells (Figure 11A) while *α*-Fe_2_O_3_-**n** NPs seemed less associated with the bacterial cells (Figure 11B). It has indeed been demonstrated previously that electrostatic interaction forces could cause positively charged NPs’ adsorption to the negatively charged surface of *E. coli* cells [46]. A similar effect was not detected in the case of *S. aureus* exposed to 100 mg/L NPs for 30 min or 24 h (Figure 11G–H). A quantitative analysis of the CLSM images revealed that significantly more *α*-Fe_2_O_3_-**p** signals were associated with *E. coli* cell signals at 30 min and *S. aureus* cell signals at 30 min and 24 h time points than *α*-Fe_2_O_3_-**n,** with the larger effect size for *E. coli* (Figure 12). The latter is in agreement with *α*-Fe_2_O_3_-**p** NPs being more toxic to *E. coli* than to *S. aureus*, as also seen in the spot test (Figure 5). CLSM images of 24 h exposed *E. coli* cells could not be analyzed due to extensive cell damage and poor fluorescence signal (data not shown). Cellular aggregate sizes of bacteria incubated with positively charged NPs and measured from CLSM maximal projections were not statistically significantly larger from nontreated controls. Therefore, we conclude that no significant heteroagglomeration [84] due to electrostatic forces between negatively charged cell surface and positively charged NPs occurred.

The fact that less negatively charged than positively charged NPs were associated per cell could be explained by the fact that a bacterial cell wall forms an electrostatic barrier, thereby limiting its interaction with negatively charged NPs [85,86]. Positively charged surface coatings on NPs, on the other hand, facilitate the cell wall breakdown and cytoplasm release [85].

One of the reasons for the different activity of NPs to *S. aureus* compared with *E. coli* could be the structural differences in the organization of bacterial cell wall. Gram-negative bacteria have a thin peptidoglycan layer (~2–3 nm) between the cytoplasmic membrane and the outer membrane; in contrast, gram-positive bacteria lack the outer membrane, but they have an outer peptidoglycan layer (about 30 nm thick) that acts as a cell protecting barrier [49,85,86], and extensive contact of the membrane with the positive charged NP is less likely to occur, even under conditions of electrostatic attraction [86].

Qu C. et al. [49] showed that surface adhesion between hematite (*α*-Fe_2_O_3_) particles and bacterial cells was initially dominated by Lifshitz van der Waals and electrostatic forces. First, the rapid formation of P−O−Fe bonds was observed, which was followed by the changes in the structure of membrane proteins. The latter caused the loss of the structural integrity of the membrane, allowing for the entrance of NPs into the bacteria cells. In addition, after the contact of hematite NPs with bacterial cells, ROS was generated on the cell’s surface, leading to cell permeabilization. The study also covered effects of different sizes of hematite particles, showing that NPs were more toxic to bacteria than microscale particles due to the stronger interfacial physicochemical interactions of nano-scale particles with the bacterial cells; according to our results, this could be explained by the negative surface charge of the studied microscale particles and the positive surface charge of the studied NPs.

### 3.7. Assessment of NP Adhesion on the Surface of Bacteria Cells by Flow Cytometry

In addition to the CLSM study, the interaction of positively and negatively charged *α*-Fe_2_O_3_ NPs with *E. coli* and *S. aureus* was quantified using the flow cytometry analysis. Bacteria were exposed to 50 mg/L *α*-Fe_2_O_3_ NPs and 70% ethanol (a positive control to yield non-viable bacteria) for 30 and 60 min, respectively. NP–cell interactions were assessed by the shift in the side-scatter (SSC) signal of the Syto9-stained cell population, which is indicative of the changes in the cell granularity [87,88]. SSC is a measure of the cell refractive index that depends on the cell granularity or internal complexity [89]. Granularity is a parameter that includes optical complexity caused by particulate material contained within the cell [90] or cell-bound nanoparticles [68].

**Figure 13:**
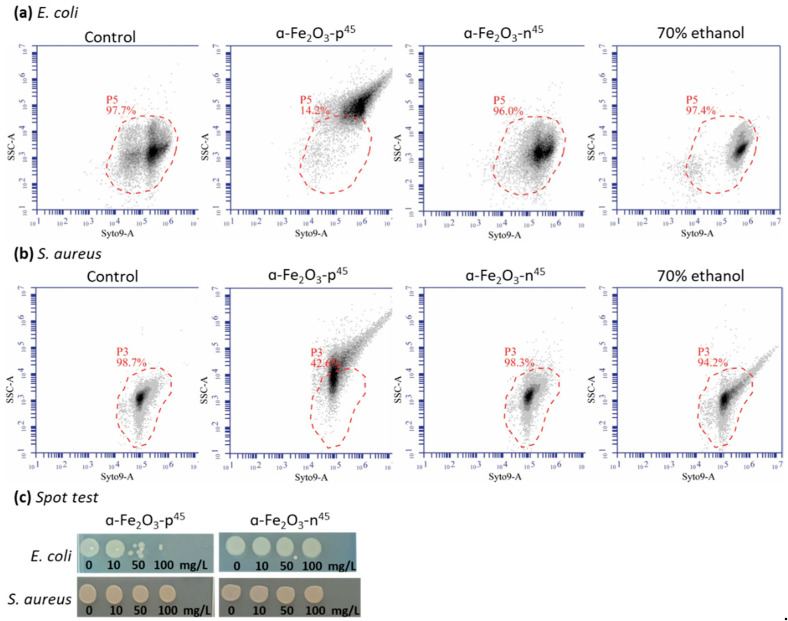
Quantification of hematite NPs binding to the bacteria *Escherichia coli* (**a**) and *Staphylococcus aureus* (**b**) by flow cytometry to determine the side-scattered (SSC) signal shift of Syto9-stained cells. Control cells-gated cell population is marked by the dashed red line. The spot test (**c**) shows that *α*-Fe_2_O_3_-**p**45 NPs at 50 mg/L were toxic to *E. coli* but not to *S. aureus*, and *α*-Fe_2_O_3_-**n**45 NPs were not toxic to both bacteria. After exposure to 70% ethanol for 60 min, the colony-forming ability of *E. coli* and *S. aureus* was not observed.

The flow cytometry analysis showed an increase in the SSC signal after exposure of *E. coli* and *S. aureus* to positively charged hematite particles (*α*-Fe_2_O_3_-**p**45) but not to the negatively charged particles (*α*-Fe_2_O_3_-**n**45) (Figure 13a,b). Moreover, the binding of positively charged NPs to bacterial cells was more prominent for *E. coli* than for *S. aureus*, i.e., 84% and 56%, respectively. Bacterial cells exposed to the 70% ethanol (a biocidal concentration) did not show the shift in SSC signal (Figure 13a, b), indicating that the changing in the cell granularity after exposing to the positively charged hematite NPs was caused by the cellular interactions and not due to the toxicity of these NPs (Figure 13c) (e.g., due to the membrane disturbance and “bubbling” of the cells). Results of the flow cytometry were consistent with the results of CLSM, showing that more positively charged NPs were associated with bacterial cells than negatively charged NPs, and this effect was larger for *E. coli*, which is also in agreement with *α*-Fe_2_O_3_-**p**45 NPs being more toxic to *E. coli* than *S. aureus.*

## 4. Conclusions

In the current study, the antibacterial activity of positively and negatively charged spherical hematite (*α*-Fe_2_O_3_) nanoparticles (NPs) with a primary size of 45 and 70 nm, respectively, was evaluated against clinically relevant bacteria *Escherichia coli* and *Staphylococcus aureus* as well as against naturally bioluminescent bacteria *Vibrio fischeri* (an ecotoxicological model organism). In the case of both types of NPs, no ROS production in abiotic or biotic conditions was observed. Thus, the positively (but not negatively) charged hematite NPs showed antibacterial properties towards gram-negative *E. coli*, probably due to the interactions of NPs and the bacterial surface (a nanobio interface). No antibacterial properties of studied NPs to gram-positive *S. aureus* were observed. These data support the tailoring of the antibacterial properties of hematite NPs towards gram-negative pathogens via the NPs’ positive surface charge. The hematite NPs, irrespective of their surface charge, were not ecotoxic, based on the *V. fischeri* assay, thus supporting the use of these NPs in wide applications where environmental consequences must be avoided.

## Figures and Tables

**Table 1:** Antimicrobial properties of hematite: literature data.

Nr.	Phase	Primary Size (nm)	Shape	ζ-potential (mV)	Method/Medium	Microorganisms	Mode of Action	Results	Coating	Reference
**1**	*α*-Fe_2_O_3_	40	-	−35 (in DI)	Agar well diffusion, MHA/sodium acetate buffer. Resazurin microdilution/MHB.	*S. aureus* *Bacillus sp.* *E. coli* *K. pneumonia* *P. aeruginosa* *S. enterica* *Vibrio sp.*	Not analyzed	Inhibition zones to all bacteria at 30 µg/mL; MIC value: *Bacillus sp.* and *Vibrio sp.* (0.98 µg/mL) > *S. aureus, P. aeruginosa*, and *S. enterica* (7.81 µg/mL) > *E. coli* (15.6 µg/mL) > *K. pneumonia* (31.25 µg/mL).	Bare	[54]
**2**	*α*-Fe_2_O_3_	25 (C) 22 (A)	Uneven	Not tested	Agar-diffusion method	*E. coli*	Not analyzed	Inhibition zone at 75 mg mL^−1^	Caralluma Fimbriata (C) Achyranthes Aspera (A)	[55]
**3**	*α*-Fe_2_O_3_	~13.5 (bare) ~6.78 (Aloe vera)	Pleomorphic morphologies	Not tested	The planktonic growth (OD measurement)/LB broth	Pristine *P. aeruginosa* low EPS *P. aeruginosa*	ALE coated NPs attachment to EPS, as a result penetration into cell and intracellular ROS activity. Bare NPs were less active, less penetration observed.	Bare NPs (1000 µg/mL): pristine ~43%, low EPS ~55% reduction; ALE NPs (1000 µg/mL): pristine ~58%, low EPS ~78% reduction.	Bare (uncoated) *Aloe vera* capped	[56]
**4**	*α*-Fe_2_O_3_	~90.46	Polygon	Not tested	Agar diffusion MHA / DI	*E. coli* *S. aureus*	Non active	No inhibition zones were observed up to a concentration of 1.5 mg/mL after 24 h of incubation	bare	[57]
**5**	*α*-Fe_2_O_3_	26 53 76 98	Spherical	28.6 57.4 55.4 67.9 (in PBS; pH 7.2)	Adsorption kinetics/PBS	*E. coli*	The toxicity may be linked more to the intake mass of NPs, rather than the intake number of NPs.	NPs concentration 100 mg/L in [Fe^3+^] NPs sorption as [Fe^3+^]: 98 nm > 76 nm > 53 nm > 26 nm	Bare	[44]
**6**	*α*-Fe_2_O_3_	41.5	Spherical	Not tested	Disk diffusion, NA/DI	*S. aureus,* *P. aeruginosa,* *E. coli* *B. subtilis*	Not analyzed	5µL of NPs from stock solution (4 mg/mL); all of the bacterial strains were revealed zone of inhibition	Uncoated *Rhus punjabensis* plant extract	[58]
**7**	*α*-Fe_2_O_3_	100	-	−48 (PBS)	Adsorption measurements/PBS	*E. coli*	Bacteria cells deformation, cell surface became hardened/stiffer, cells shifted to a more-negative surface charge. Sorption of hematite.	Adhesion and sorption of NPs (100 mg/L) by *E.coli*	Bare	[45]
**8**	*γ*-Fe_2_O_3_	7–10	Quasi-spherical and cuboidal shape	−15.8(DMSO) −26.5(H_2_O) −21.5(PBS; pH: 7) +22.4(PBS; pH: 5) −21.6(PBS; pH: 9)	Well diffusion, MHA/DMSO	*K. pneumonia* *S. epidermidis* *B. subtilis* *E. coli* *P. aeruginosa* *A. flavus* *F. solani* *Mucor sp.* *A. fumigates* *A. niger*	Not analyzed	All of the bacterial strains were revealed zone of inhibition in a concentration dependent manner (4 mg/mL–250 mg/mL). *A. niger* and *A. fumigates* were not inhibited (500 µg/mL)	Bare	[59]
**9**	*α*-Fe_2_O_3_	~34.1–35.5	Quasi-spherical	Not tested	Agar well diffusion, MHA/DMSO	*E. coli* *S. aureus*	Not analyzed	All of the bacterial strains were revealed zone of inhibition (1 mg/mL); bigger zones were observed against gram-positive bacteria.	Bare	[60]
**10**	*α*-Fe_2_O_3_	90 (AF) 97 (DF) 80 (GF)	Dumbbell nature	Not tested	Agar well diffusion, MHA	*A. hydrophila* *E. coli* *S. aureus* *P. aeruginosa* *E. faecalis* *S. pyogenes*	Not analyzed	*A. hydrophila* and *E. coli* at 50 mg/mL were revealed zone of inhibition.	agarose (AF) dextran (DF) gelatin (GF)	[61]
**11**	*α*-Fe_2_O_3_	2 43 85 540	Spherical or rhombohedral (540 nm irregular shape)	Not tested	Biofilm formation/Iron deficient media M9	*P. aeruginosa*	Not analyzed	Increase the biofilm formation (10 µg/mL)	Bare	[48]
**12**	*γ*-Fe_2_O_3_	-	Spherical	Not tested	Agar well diffusion, NA/0.9% NaCl	*E. coli* *P. aeruginosa* *S. aureus* *S. pyogenes* *C. albicans*	Antimicrobial activity was not observed	Inhibition zones were not observed (1.5, 5.0 and 10 mg/mL)	Bare	[47]
**13**	*α*-Fe_2_O_3_	10 80 1000	Not mentioned	18.73 (10 nm) 17.53 (80 nm) −15.57 (1 µm) (in 0.15M NaCl; pH 7)	Adhesion measurements/0.15 M NaCl	*P. putida* *B. subtilis*	(1) Adhesion (10 nm and 80 nm) onto bacterial cells; (2) chemical bonds form, and changes in the structure of membrane proteins result in the loss of the structural integrity of the membrane; (3) NPs migration into the cells and surface-derived ROS production;	The attraction for bacterial cells increased several folds on the 10 nm NPs. The significant rupture of *P. putida* cell walls and the internalization of 10 nm NPs were observed.	Bare	[49]
**14**	*α*-Fe_2_O_3_	10.04	Spherical	+39 (media not mentioned)	Broth dilution assay, OD_600_ measurements/ nutrient media Petri plate-based standard bacterial strains were spread on nutrient-rich agar already augmented by NPs	*E. coli* *V. cholerae*	Not analyzed	MIC 150 µg/mL for both bacteria. 150 µg/mL caused 19% and 60% inhibition of *V. cholerae* and *E. coli*, respectively	Bare	[21]
15	*α*-Fe_2_O_3_	50–100	Irregular shape	Not tested	Qualitative standard ASTM-E2149-0/PBS	*S. aureus* *E. coli* *P. aeruginosa*	Surface zero charge of the membrane	5 wt.% NPs in membrane Average reduction of ~69% for *E. coli*, of ~78% for *P. aeruginosa*, ~56% for *S. aureus*	Cellulose acetate porous membrane	[62]

Mueller–Hinton agar—MHA; Mueller–Hinton broth—MHB; optical density—OD; Luria–Bertani—LB; extracellular polymeric substances—EPS; phosphate buffered saline—PBS; dimethyl sulfoxide—DMSO; nutrient broth agar—NA; *Klebsiella pneumonia*—*K. pneumonia*; *Pseudomonas aeruginosa*—*P. aeruginosa; Salmonella enterica*—*S. enterica; Bacillus subtilis*—*B. subtilis*; *Staphylococcus epidermidis*—*S. epidermidis; Aspergillus flavus*—*A. flavus*; *Fusarium solani*—*F. solani*; *Aspergillus fumigates*—*A. fumigates*; *Aeromonas hydrophila*—*A. hydrophila*; *Aspergillus niger*—*A. niger*; *Enterococcus faecalis*—*E. faecalis*; *Streptococcus pyogenes*—*S. pyogenes*; *Streptococcus pyogenes*—*S. pyogenes*; *Candida albicans*—*C. albicans*; *Pseudomonas putida*—*P. putida*; *Vibrio cholerae*—*V. cholerae.*

**Table 2:** Designation of synthesized hematite (*α*-Fe_2_O_3_) NPs based on their primary size and surface coating/charge.

Fe(III) Nitrate’s Concentration Used for the Synthesis of Hematite *α*-Fe_2_O_3_ NPs	0.05 M	0.1 M
Positively charged uncoated/bare hematite NPs, 45 or 70 nm primary size	*α*-Fe_2_O_3_-**p**45	*α*-Fe_2_O_3_-**p**70
Negatively charged citrate-coated hematite NPs, 45 or 70 nm primary size	*α*-Fe_2_O_3_-**n**45	*α*-Fe_2_O_3_-**n**70

**Table 3:** Hydrodynamic size, ζ-potential, and solubility of hematite NPs after 0 h and 24 h incubation in the testing media used for the antibacterial assays.

	ζ-potential, mV		Pdl		Hydrodynamic Size, nm		Solubilized Fe from 100 mg/L *α*-Fe_2_O_3_ NPs, ppm (TXRF)	
**In DI water (testing medium for the assays using *E. coli* and *S. aureus*)**								
**Samples**	**0 h**	**24 h**	**0 h**	**24 h**	**0 h**	**24 h**	**30 min**	**24 h**
*α*-Fe_2_O_3_-**p**45	+38.8	+42.1	0.20	0.21	116	125	0.096	0.06
*α*-Fe_2_O_3_-**p**70	+40.2	+33.3	0.25	0.23	147	137	0.11	0.1
*α*-Fe_2_O_3_-**n**45	−33.7	−44.7	0.21	0.20	100	108	0.04	0.07
*α*-Fe_2_O_3_-**n**70	−44.7	−44.6	0.19	0.19	110	104	0.05	0.07
**In 2% NaCl (testing medium for the *V. fischeri* assay)**								
**Samples**	**0 h**	**24 h**	**0 h**	**24 h**	**0 h**	**24 h**	30 min *	24 h *
*α*-Fe_2_O_3_-**p**45	+37.5	−0.6	0.44	0.44	1801	3927	-	-
*α*-Fe_2_O_3_-**p**70	+45.7	−3.4	0.32	0.47	917	2535	-	-
*α*-Fe_2_O_3_-**n**45	−25.3	−7.9	0.43	1.00	935	4014	-	-
*α*-Fe_2_O_3_-**n**70	−16.8	−18.5	0.38	0.76	2347	3238	-	-

* not tested, PdI—polydispersity index.

## Data Availability

The data presented in this study are available on request from the corresponding authors.

## Supplementary Materials

The following are available online at https://www.mdpi.com/2079-4991/11/3/652/s1, Table S1: pH of the samples at different NPs concentrations in DI water and 2% NaCl. Figure S1: TEM images of the *α*-Fe_2_O_3_ NP. Table S2: pH values—*V. fischeri* in 96-well plate after 24 h incubation. Figure S2: Agar Diffusion test visualization—*Staphylococcus aureus*. Figure S3: Generation of ROS measured in biotic conditions after 24 h of NP incubation with *E. coli* and *S. aureus* and 30 min after DCF-DA dye adding. Figure S4: Single-channel and multi-channel projection images for 30 min and 24 h time points for *E. coli*, Figure S5: Single-channel and multi-channel projection images for 30 min and 24 h time points for *S. aureus*.
