# Supplementary material for: Antibacterial Activity of Positively and Negatively Charged Hematite (α-Fe2O3) Nanoparticles to Escherichia coli, Staphylococcus aureus and Vibrio fischeri
Source: Nanomaterials (Basel). 2021 Mar 8;11(3):652. doi: 10.3390/nano11030652 (PMC7999532; doi:10.3390/nano11030652)
Supplement: Supplementary file 1 [file nanomaterials-11-00652-s001.pdf]

nanomaterials

Supplementary Materials.

Antibacterial Activity of Positively and Negatively Charged Hematite (α-Fe_2O_3) Nanoparticles to *Escherichia coli, Staphylococcus aureus* and *Vibrio fischeri*

Svetlana Vihodceva [1,*], Andris Šutka [1], Mariliis Sihtmäe [2], Merilin Rosenberg [2,3], Maarja Otsus [2], Imbi Kurvet [2], Krišjanis Šmits [4], Liga Bikše [4] and Anne Kahru [2,5,*] and Kaja Kasemets [2]

[1] Research Laboratory of Functional Materials Technologies, Faculty of Materials Science and Applied Chemistry, Riga Technical University, Paula Valdena 3/7, LV-1048 Riga, Latvia; Andris.Sutka@rtu.lv
[2] Laboratory of Environmental Toxicology, National Institute of Chemical Physics and Biophysics, Akadeemia tee 23, 12618 Tallinn, Estonia; Mariliis.Sihtmae@kbfi.ee (M.S.); Merilin.Rosenberg@kbfi.ee (M.R.); Maarja.Otsus@kbfi.ee (M.O.); Imbi.Kurvet@kbfi.ee (I.K.); Kaja.Kasemets@kbfi.ee (K.K.)
[3] Institute of Chemistry and Biotechnology, Tallinn University of Technology, Akadeemia tee 15, 12618 Tallinn, Estonia
[4] Institute of Solid State Physics, University of Latvia, Kengaraga 8, LV-1063 Riga, Latvia; smits@cfi.lu.lv (K.S.); lbikshe@gmail.com (L.B.)
[5] Estonian Academy of Sciences, Kohtu 6, 10130 Tallinn, Estonia
* Correspondence: Svetlana.Vihodceva@rtu.lv (S.V.); Anne.Kahru@kbfi.ee (A.K.)

Cultivation of Bacteria.

E. coli MG 16551 and *S. aureus* ATCC 6538 were grown overnight in LB medium (yeast extract (LabM, UK) 5 g/L; tryptone (LabM, UK) 10 g/L; NaCl (Sigma-Aldrich, USA) 5 g/L). Overnight cultures were diluted 1:50 in fresh LB medium and cultivated up to the exponential growth phase (optical density (OD) at 600 nm = 0.6 as measured by Jenway 6300 spectrophotometer (UK), using 1 cm path length cuvette) on the orbital shaker (Thermo Scientific Forma Orbital Shaker 420, USA) at 200 rpm, 30 °C. Then, the bacterial suspension was washed twice with DI water by centrifugation at 7500 rpm for 5 min and finally suspended in DI adjusting the culture density to the OD_{600nm} = 0.1 (2×10^7 CFU/mL; CFU – cell forming unit determined by the plating and counting of colonies on the LB agar plates).

pH of the Samples at Different NPs Concentrations in DI water and 2% NaCl Testing Media.

Table S1. pH of the samples at different NPs concentrations in DI water and 2% NaCl.

**Samples pH at Different NPs Concentrations**				
**In DI water (DI water pH 5.8) (a testing medium for the assays using *E. coli* and *S. aureus*)**				
**NPs, mg/L**	**α-Fe_2O_3-p45**	**α-Fe_2O_3-p70**	**α-Fe_2O_3-n45**	**α-Fe_2O_3-n70**
1000	6.69	6.60	6.41	6.53
500	6.49	6.41	6.48	6.58
250	5.99	6.36	6.33	6.40
100	6.09	6.28	6.10	6.02
10	6.22	6.18	6.05	6.26
1	6.03	6.08	5.92	6.00
**In 2% NaCl (2% NaCl pH 5.95)** (a testing medium for the *V. fischeri* assay)				
1000	6.42	7.1	6.61	6.66
500	6.30	6.99	6.48	6.47
250	6.15	6.67	6.28	6.29

100	5.95	6.31	6.12	6.07
10	5.83	5.98	5.91	5.95
1	5.83	5.80	5.88	5.89

TEM.

The TEM analysis shows small spherically shaped NPs which size increase with higher concentration of precursor, with an average size of uncoated M α-Fe_2O_3-**p**45 - 43 ± 7 nm and α-Fe_2O_3-**p**70 - 63 ± 12 nm (Figure 1A–B) and citrate coated α-Fe_2O_3-**n**45 - 44 ± 8 nm and α-Fe_2O_3-**n**70 - 63 ± 10 nm (Figure 1C–D).

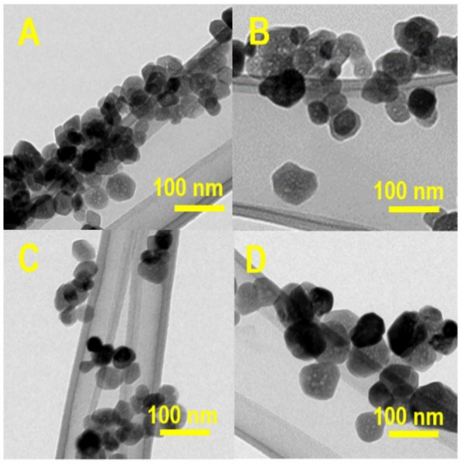

Figure S1. TEM images of the α-Fe_2O_3 NP: **A)** α-Fe_2O_3-**p**45, **B)** α-Fe_2O_3-**p**70, **C)** α-Fe_2O_3-n45 and **D)** α-Fe_2O_3-**n**70.

***Vibrio fischeri* Flash Assay pH Values.**

Table S2. pH values - *V. fischeri* in 96-well plate after 24 h incubation.

**pH in 96-Well with *V. fischeri* after 24 h Incubation**				
**Control (2% NaCl pH 6.71) (a testing medium for the *V. fischeri* assay)**				
**NPs, mg/L**	**α-Fe_2O_3-p45**	**α-Fe_2O_3-p70**	**α-Fe_2O_3-n45**	**α-Fe_2O_3-n70**
1000	6.24	6.24	6.58	6.88
500	6.31	6.38	6.55	6.92
250	6.33	6.36	6.80	6.92
100	6.34	6.39	6.82	6.92
10	6.39	6.32	6.80	6.90
1	6.36	6.35	6.80	6.91

Agar Diffusion Test.

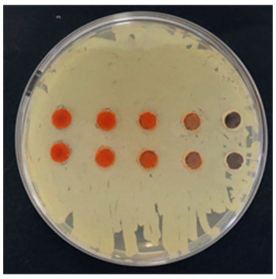 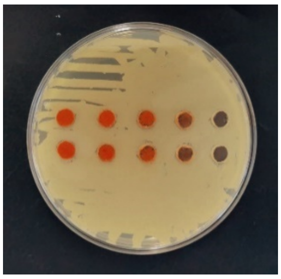

.

(**a**) (**b**)

Figure S2. Agar Diffusion test visualization - *Staphylococcus aureus*: **(a)** α-Fe_2O_3-**p**, **(b)** α-Fe_2O_3-**n**.

ROS Generation in Biotic Condition after 24 h of NP + Bacteria Incubation.

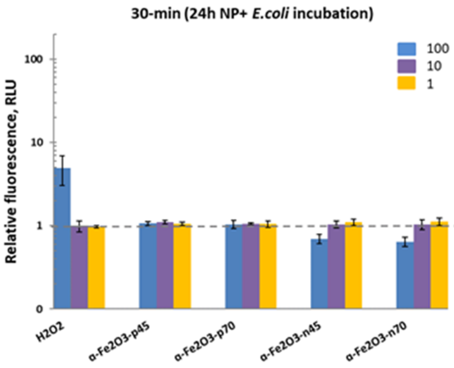

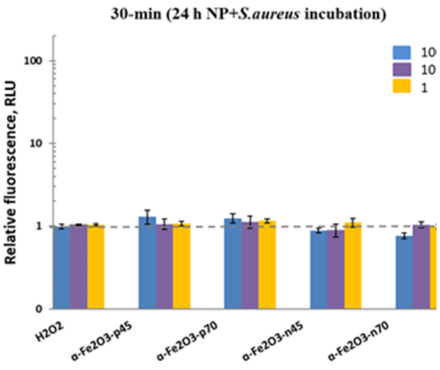

(a) **(b)**

Figure S3. Generation of ROS measured with fluorescent dye DCF-DA in biotic conditions after 24 h of NP incubation with *Escherichia coli* **(a)** and *Staphylococcus aureus* **(b)** and 30 min after DCF-DA dye adding.

CLSM Single-Channel and Multi-Channel Projection Images.

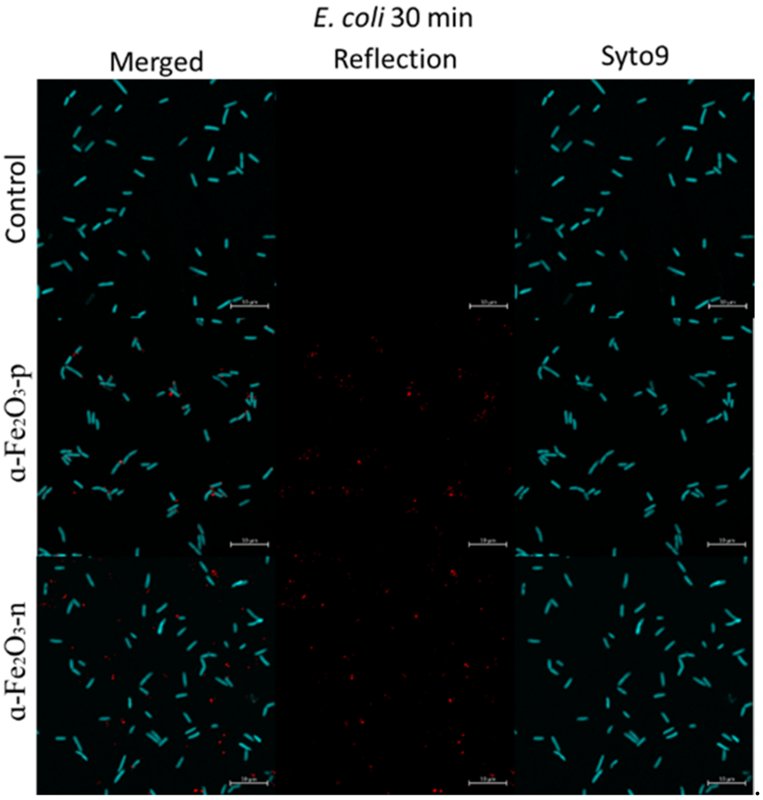

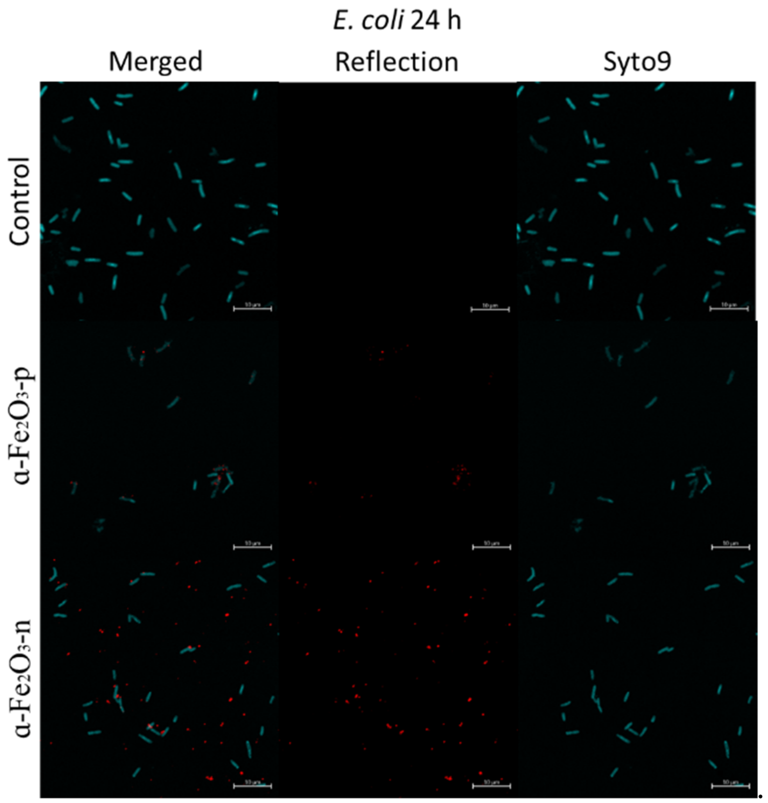

Figure S4. Single-channel and multi-channel projection images for 30 min and 24 h time points for *Escherischia coli.*

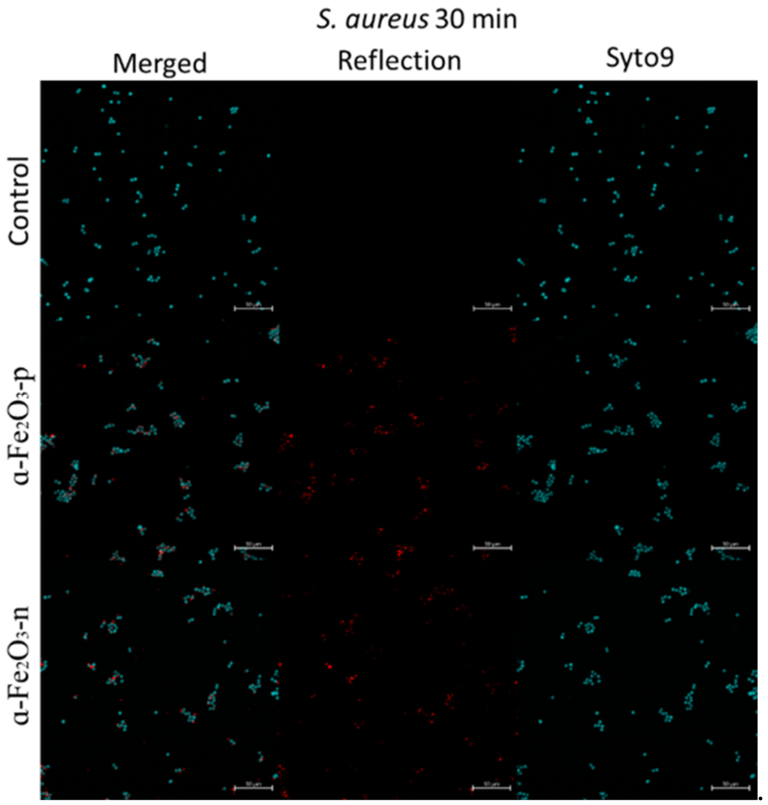

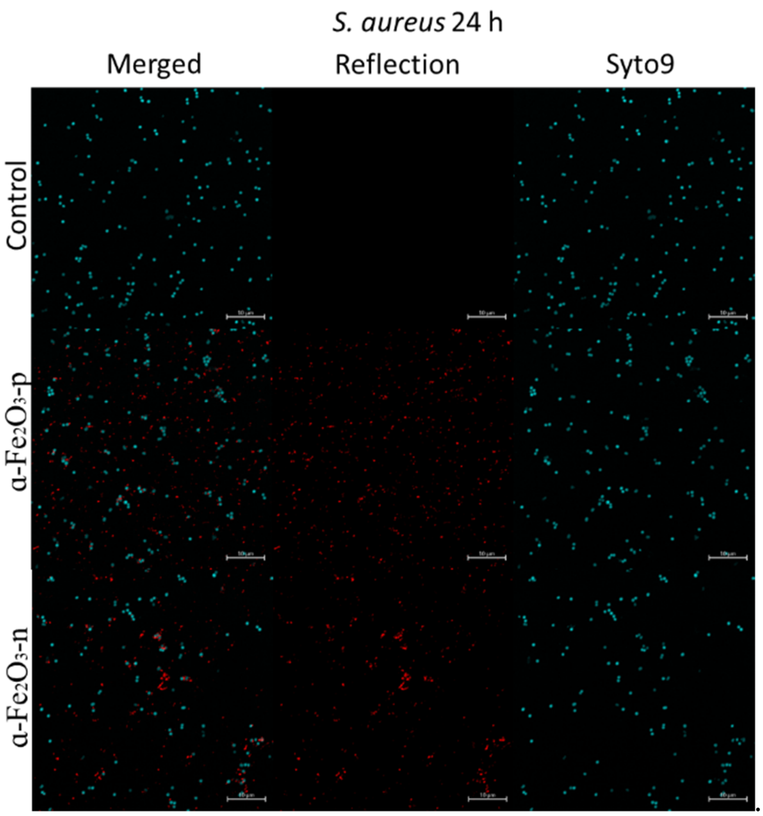

Figure S5. Single-channel and multi-channel projection images for 30 min and 24 h time points for *Staphylococcus aureus*.
